# Dynamic Stability of Tensegrity Structures—Part II: The Periodic External Load

**DOI:** 10.3390/ma16134564

**Published:** 2023-06-24

**Authors:** Paulina Obara, Justyna Tomasik

**Affiliations:** Faculty of Civil Engineering and Architecture, Kielce University of Technology, Al. Tysiąclecia Państwa Polskiego 7, 25-314 Kielce, Poland; paula@tu.kielce.pl

**Keywords:** tensegrity structures, initial prestress forces, infinitesimal mechanism, parametric resonance, instability region

## Abstract

The paper contains a parametric analysis of tensegrity structures subjected to periodic loads. The analysis focuses on determining the main region of dynamic instability. When load parameters fall within this region, the resulting vibration amplitudes increase, posing a risk to the durability of structures. The study considers structures built using commonly used modules. The influence of the initial prestress on the distribution of the instability regions is examined. Additional prestress can significantly reduce the extent of instability regions, potentially narrowing them by up to 99%. A nondimensional parameter is introduced to accurately assess changes in the extent of the instability region. A geometrically non-linear model is employed to evaluate the behavior of the analyzed structures.

## 1. Introduction

Stability refers to the ability of a system to maintain its configuration and resist any tendency to undergo significant changes or deformations under the influence of external loads. This is directly related to the type of equilibrium. The equilibrium of a deformed configuration can be stable or unstable. In the case of periodic dynamic loads, the analysis of the permanent equilibrium can be equated with the study of the stability of motion. Assuming a system with scleronomic constraints, its motion is an oscillation around the static equilibrium position. If a small perturbation induces a new motion with limited solutions, the original motion is static. The instability of the induced motion is the same as the instability of the original motion. It is a case of dynamic instability or parametric resonance. Unstable movements are dangerous in terms of the durability of the structure. The study and assessment of the stability of system movements caused by various types of excitation is called the dynamic stability (Lyapunov stability) [1,2,3]. Dynamic stability, or what Bolotin referred to as “dynamic instability” [4], involves determining the resonant frequencies of periodic excitations and mapping out stable and unstable regions using Ince–Strutt maps (parametric resonance regions). From the perspective of the physical interpretation, when the load parameters fall within the defined limits of instability, a structure undergoes vibrations characterized by an amplification of amplitude. The Ince–Strutt maps are obtained by solving the equations of motion, which, in the case of structures subjected to periodic load, are generally expressed by a nonhomogeneous Mathieu–Hill equation [5]. This is a second-order linear differential equation that describes the behavior of a parametrically forced system. It is named after the French mathematician Émile Mathieu and the British mathematician George William Hill. The Mathieu–Hill equation captures the essential characteristics of parametric resonance, where the behavior is influenced by the interplay between the natural frequency and the modulation amplitude. The equation allows for the analysis of stability, resonance regions, and the determination of stable and unstable solutions. The stable regions correspond to finite solutions of the Mathieu–Hill equations, while the unstable regions correspond to solutions that increase indefinitely in time [3]. The stability charts of the Mathieu–Hill equations published in the literature are usually calculated using various methods, i.e., harmonic balance method [4,6,7,8,9,10,11], Galerkin method [11,12], multiscale methods [13], averaging method [14,15], discrete singular convolution [16], homotopy perturbation method [17], and small parameter method (Poincaré method) [3,11]. There is a wealth of literature available on parametric vibrations that comprehensively addresses all fundamental aspects of the subject [3,4,6,7,8,18,19]. However, the dynamic instability of elastic structures, from analytical, numerical, and experimental perspectives, is an interesting and popular topic [12,13,20,21], and many issues still remain unsolved. A comprehensive overview of current research interests related to the dynamic instability of elastic structures is presented in various studies, including reference [22]. One unresolved issue concerns tensegrity systems that exhibit infinitesimal mechanisms, which are stabilized by a self-balancing system of internal forces known as the self-stress state or initial prestress forces. Based on our current recognition, researchers have carried out many studies on tensegrity systems (see Part 1 [23]). Several recent papers have explored the dynamics of tensegrity structures, including references [24,25,26]. These studies aim to determine the natural and free frequencies of vibrations in such structures. However, the analysis of dynamic stability, as understood in terms of the Bolotin approach [4,6], has not been conducted. This aspect is often mistakenly conflated with issues related to impulse loads [27]. Examining the behavior of structures under the influence of periodic dynamic loads is crucial for ensuring the durability of tensegrity structures. They are distinguished by an additional parameter, the initial prestress, which has a significant impact on the shape and extent of instability regions. Considering the aforementioned points, it seemed reasonable to explore the subject of dynamic analysis, particularly focusing on the analysis of dynamic stability in tensegrity structures.

This research addresses a gap in the existing literature by examining the response of tensegrity structures to periodic loads. The primary objective is to determine the resonant frequencies of periodic excitations and identify unstable regions as a function of the initial prestress. The study employs the harmonic balance method as a tool for this analysis. The proposed methodology offers the advantage of using a well-established approach to identify instability regions in distinct structures. This study intends to describe the behavior of tensegrity structures considered in the previous part [23], and together with Part 1, it is a complete procedure to investigate the behavior of the tensegrity structure under external loads. To validate the proposed approach, the behavior of a simple two-element truss is considered. Although this structure is not a tensegrity itself, its behavior accurately mirrors that of tensegrity structures. As a result, it enables the explicit determination of the influence of the initial prestress level on unstable regions, as presented in this study. The research focuses on tensegrity structures constructed using modified Simplex and Quartex modules. In this study, a linear connection is examined, encompassing various methods of connecting the modules and considering different support conditions. A nondimensional parameter λ is introduced for the quantitative assessment. This parameter expresses the ratio of the area of the unstable region at the *i*-th level of initial prestress to the area of the unstable region at the minimum level of initial prestress. It is a measure of changes in the area of the instability region, i.e., the effect of the initial prestress level. The analysis in this study employs a nonlinear approach, assuming the hypothesis of large displacements [23,28,29]. The provided description is adequate for analyzing both planar and spatial lattice structures, including tensegrity structures, under geometrically nonlinear and physically linear conditions.

The paper is organized as follows: Section 2 describes the Mathieu–Hill equations for the tensegrity structures and one of the most popular methods for solving this equation, i.e., the harmonic balance method. In Section 3, the solution procedures are demonstrated using a simple two-element structure that exhibits a self-stress state and an infinitesimal mechanism. Furthermore, the results for tensegrity structures built with the modified Simplex and Quartex modules are given in Section 4. Lastly, in Section 5, several conclusions are drawn based on the findings of the study. The calculation procedure was implemented using the Mathematica environment. This procedure enables the generation of diagrams that illustrate the locations of instability regions as a function of the dynamic force applied and the vibration frequency of the analyzed structures. Moreover, it takes into consideration the impact of the initial prestress on the instability regions.

## 2. Methods of Analysis

In the analysis of tensegrity structures described in the paper, a discrete formulation based on finite element methods is employed. A tensegrity system is characterized as a spatial, lightweight structure composed of compressed struts and tensioned cables. The structure is an n-element truss with m degrees of freedom, where each element is labeled by e=1,2,…,n. The displacement of the structure is represented by the vector $q\left(\in {\mathbb{R}}^{m\times 1}\right)$. Tensegrity structures are assembled in a self-balanced manner, meaning that in the absence of external loads, there is an equilibrium between the forces in the struts and cables. This equilibrium configuration of internal forces is referred to as the “self-stress state” ${(y}_{S})$ and its determination is outlined in reference [23]. Once the self-stress state is defined, the geometric stiffness matrix $K_{G}(S)\left(\in {\mathbb{R}}^{m\times m}\right)$ can be constructed, where the longitudinal forces S depend on the self-stress state $y_{S}$ and the initial prestress level $\left({S=y}_{S}S\right)$). The self-stress state stabilizes one or more infinitesimal mechanisms, which are inherent features of tensegrity structures. Furthermore, tensegrity structures exhibit stiffening behavior when subjected to external loads. The applied load induces displacements according to the form of the infinitesimal mechanism, resulting in additional prestress in the structure. Tensile forces generate further tension in the cables, while compressive forces arise in the struts. The component of the stiffness matrix related to the axial forces N resulting from the initial load P is denoted as $K_{GN}(N)\left(\in {\mathbb{R}}^{m\times m}\right)$.

Furthermore, like any discrete structure, it is described by the following matrices: $K_{L}\left(\in {\mathbb{R}}^{m\times m}\right)$ is the linear stiffness matrix, $M\left(\in {\mathbb{R}}^{m\times m}\right)$ represents the consequent matrix of masses, and $K_{NL}\left(q\right)\left(\in {\mathbb{R}}^{m\times m}\right)$ is the non-linear displacement stiffness matrix. The explicit forms of these matrices for the tensegrity element can be found, for instance, in reference [28]. To evaluate the behavior of tensegrity structures, a geometrically non-linear model is used [3,28,30]. The non-linear theory of elasticity in the Total Lagrangian (TL, Lagrange’s stationary description) approach is adopted as a basis for formulating the tensegrity lattice equations.

The main purpose is the determination of resonant frequencies of periodic extortions and instable regions (Ince–Strutt maps) as a function of initial prestress. The determination of the Ince–Strutt maps with stable and unstable regions (parametric resonance regions) relies on the determination of the resonant frequencies of the periodic load:

(1) $$P\left(t\right)=P+P_{t}\Phi \left(t\right),\;$$

where P is the constant part of the load, $P_{t}$ is the amplitude of the periodic force, and $\Phi \left(t\right)$ is the periodic function that describes the time variation of the force. The unstable regions, or parametric resonance regions, occur at specific frequencies. These frequencies are determined by the equation:

(2) $$\Omega =\frac{\Omega_{i}}{k}\;\mathrm{or}\;\Omega =\frac{\Omega_{i}\pm \Omega_{j}}{2k};\;k=1,2,\ldots ;\;i\neq j,$$

where Ω is the resonant frequency and $\Omega_{i}$ and $\Omega_{j}$ are the free frequencies of the structure under the influence of the constant load P. In the case of periodic resonances, the unstable regions occur at frequencies $\frac{\Omega_{i}}{k}$, while in combined resonances, the unstable regions occur at frequencies $\frac{\Omega_{i}\pm \Omega_{j}}{2k}$. Of particular interest are the main instability regions, which correspond to the first-order periodic resonances (k=1).

### 2.1. Mathieu–Hill Equations

The equation of motion in the case of structures subjected to periodic load (1) is expressed by a nonhomogeneous Mathieu–Hill equation:

(3) $$M\ddot{q}\left(t\right)+K_{L}q\left(t\right)+\left[1+\upsilon \Phi \left(t\right)\right]K_{G}q\left(t\right)=0;\;\upsilon =\frac{P_{t}}{P},\;$$

where $\ddot{q}\left(\in {\mathbb{R}}^{m\times 1}\right)$ is the acceleration vector and υ is the pulsatility index. Equation (3) describes the free vibrations under the influence of the initial load (1), taking into account the geometric stiffness matrix. It should be noted that in the case of tensegrity structures, the geometric stiffness matrix consists of two parts:

(4) $$K_{G}=K_{G}\left(S\right)+K_{GN}\left(N\right).$$

The calculation of the second part of the geometric stiffness matrix requires a nonlinear analysis:

(5) $$\left[{K_{L}+K}_{G}(S)+K_{NL}\left(q\right)\right]q=P,$$

where $P\left(\in {\mathbb{R}}^{m\times 1}\right)$ is a time-independent external load vector. The non-linear Equation (5), which is solved through an incremental-iterative analysis of large displacement gradients (further details can be found in Part 1 [23]), subsequently results in real normal forces in the elements $N^{e}$:

(6) $$N^{e}=E^{e}A^{e}\varepsilon^{e}\sqrt{1+2\varepsilon^{e}};\;\varepsilon^{e}=\frac{1}{2}\frac{{\left(l_{1}^{e}\right)}^{2}-{\left(l^{e}\right)}^{2}}{{\left(l^{e}\right)}^{2}},$$

where $E^{e}$ is the Young modulus of an element, $A^{e}$ is the cross-sectional area of an element, $l^{e}$ is the length of the element in the initial configuration, and $l_{1}^{e}$ is the length of the element in the actual configuration:

(7) $$l_{1}^{e}={\left(\Delta_{u_{2}}\right)}^{2}\sqrt{{\left(\Delta_{u_{2}}\right)}^{2}+{\left(\Delta_{u_{3}}\right)}^{2}+{\left(l^{e}+\Delta_{u_{1}}\right)}^{2}},$$

where $\Delta_{u_{i}}=q_{i}^{2}-q_{i}^{1}(i=1,2,3)$ represents the displacement increments between nodes two ${(q}_{i}^{2})$ and one $\left(q_{i}^{1}\right)$.

The nonlinear parametric equation of motion (3) is known as the Hill equation. Considering a harmonic periodic force with frequency θ:

(8) $$\Phi \left(t\right)=\cos\left(\theta t\right);\;\theta =\frac{2\pi }{T},$$

the Hill Equation (3) can be expressed as:

(9) $$M\ddot{q}\left(t\right)+\left[K_{L}+\left(1+\upsilon cos\left(\theta t\right)\right)K_{G}\right]q\left(t\right)=0$$

and is referred to as the Mathieu equation. To solve the nonlinear equations of motion (9), the commonly used approach is the harmonic balance method.

### 2.2. Harmonic Balance Method

The harmonic balance method is a powerful technique used to solve nonlinear equations, particularly in the context of periodic systems. It offers several advantages over other existing methods. The primary benefits of the harmonic balance method encompass its efficiency, accuracy, applicability in frequency response analysis, capability for nonlinear parameter identification, facilitation of system optimization and control, and operation within the frequency domain. To determine the first instability region, the Fourier series with a period of 2T can be employed:

(10) $$q\left(t\right)=\sum_{k=1,3,5}^{\infty }\left(a_{k}sin\frac{k\theta t}{2}+b_{k}cos\frac{k\theta t}{2}\right),$$

where $a_{k}$ and $b_{k}$ are constant coefficients. By substituting Equation (10) into Equation (9), a linear combination of trigonometric functions is obtained:

(11) $$A_{1}sin\frac{\theta t}{2}+B_{1}cos\frac{\theta t}{2}+A_{3}sin\frac{3\theta t}{2}+B_{3}cos\frac{3\theta t}{2}+A_{5}sin\frac{5\theta t}{2}+\cdots =0.$$

The coefficients $A_{i}$ and $B_{i}$ are derived by balancing the terms with the appropriate harmonics:

(12) $$\begin{array}{c} A_{1}=C_{1}a_{1}-Da_{1}+Da_{3},\;\;\;\;B_{1}=C_{1}b_{1}+Db_{1}+Db_{3},\\ A_{3}=Da_{1}+C_{3}a_{3}+Da_{5},\;\;\;\;B_{3}=Db_{1}+C_{3}b_{3}+Db_{5},\\ A_{5}=Da_{3}+C_{5}a_{5}+Da_{7},\;\;\;\;B_{5}=Db_{3}+C_{5}b_{5}+Db_{7}, \end{array}$$

where:

(13) $$D=\frac{1}{2}\upsilon K_{G},\;\;C_{k}=K_{L}+K_{G}-\frac{k^{2}\theta^{2}}{4}M;\;\;k=1,3,5,\ldots .$$

The satisfaction of Equation (11) for each t leads to the following condition:

(14) $$A_{1}=B_{1}=A_{3}=B_{3}=A_{5}=\cdots =0.$$

This condition, in turn, gives rise to linearly separated homogeneous systems with an infinite number of equations and unknowns, $a_{k}$ and $b_{k}$. Non-zero solutions exist when the following conditional equation is satisfied:

(15) $$Det\left\{\begin{array}{cccc} K_{L}+K_{G}\pm \frac{1}{2}\upsilon K_{G}-\frac{1}{4}\theta^{2}M & \frac{1}{2}\upsilon K_{G} & 0 & \cdots \\ \frac{1}{2}\upsilon K_{G} & K_{L}+K_{G}-\frac{9}{4}\theta^{2}M & \frac{1}{2}\upsilon K_{G} & \cdots \\ 0 & \frac{1}{2}\upsilon K_{G} & K_{L}+K_{G}-\frac{25}{4}\theta^{2}M & \cdots \\ \cdots & \cdots & \cdots & \cdots \end{array}\right\}=0.$$

Considering the determinant of the first degree in Equation (15), it is sufficient to obtain the boundaries of the main instability regions:

(16) $$det\left\{K_{L}+\left(1\pm \frac{1}{2}\upsilon \right)K_{G}-\frac{\theta^{2}}{4}M\right\}=0.$$

Solving Equation (16) allows determining the main instability regions (Ince–Strutt maps) in the parameter plane:

(17) $$\upsilon =\frac{P_{t}}{P},\;\eta =\frac{\theta }{2\pi },\;$$

where η is the resonant frequency of external load.

In contrast to conventional cable-strut frameworks, tensegrity structures are characterized by an additional parameter, the initial prestress S. This parameter affects the shape and range of instability regions. In order to measure changes in the instability region and assess the effect of the initial prestress level, the following non-dimensional parameter is introduced:

(18) $$\lambda =\frac{A_{\eta }(S_{i})}{A_{\eta }(S_{min})}\;,$$

where $A_{\eta }\left(S_{i}\right)$ is an area of instability region at the i-th initial prestress level, while $A_{\eta }(S_{min})$ is an area at the minimum initial prestress level.

## 3. Behavior of Structures Characterized by a Self-Stress State and Infinitesimal Mechanism

**Figure 1:**
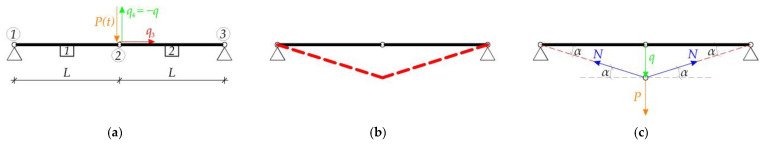
(**a**) Two-element truss, (**b**) infinitesimal mechanism, (**c**) equilibrium of node 2.

To validate the proposed methodology, the behavior of a simple two-element (n=2) structure is examined (Figure 1a). The elements are characterized by the Young modulus E, the cross-sectional area A, the density ρ, and the length L. The structure has two degrees of freedom (m=2)—$q={\left[\begin{array}{cc} q_{3} & q_{4} \end{array}\right]}^{T}$ and exhibits one self-stress state S characterized by self-stress forces $N^{1}=N^{2}=S$ and one infinitesimal mechanism (Figure 1b). To analyze the dynamic behavior of the structure, a periodic force (1) is applied. This force consists of the constant part P, the amplitude $P_{t}$, and the frequency θ (as defined in (8)). The force is applied to node 2 in the vertical direction:

(19) $$P\left(t\right)=P+P_{t}\cos\left(\theta t\right)$$

Due to the symmetry of the structure and the load, the displacement $q_{3}$ is equal to zero. Therefore, the structure has only one degree of freedom $\left(m=1\right)$, represented by $q={-q}_{4}$. The characteristics related to mass $m_{\rho }$ and stiffness $k(k=k_{L}+k_{G}\left(S\right)+k_{GN}\left(N\right)+k_{NL})$ are as follows:

(20) $$m_{\rho }=\frac{2\rho AL}{3};\;k_{L}=0;\;k_{G}\left(S\right)=\frac{2S}{L};\;k_{GN}\left(N\right)=\frac{2N(P)}{L};\;k_{NL}=\frac{EA}{L^{3}}q^{2}.$$

In the case of structures with one degree of freedom, the first natural frequency of vibration is described by the formula:

(21) $$f_{1}=\frac{1}{2\pi }\sqrt{\frac{k}{m_{\rho }}};\;{k=k}_{G}\left(S\right)+k_{GN}\left(N\right).$$

It should be noted that on the basis of Equation (21), the natural $f_{1}\left(0\right)$ and the free frequencies $f_{1}\left(P\right)$, which depend on the constant part P of the load, can be calculated as follows:

(22) $$f_{1}\left(0\right)=\frac{1}{2\pi }\sqrt{\frac{3S}{\rho AL^{2}}},\;f_{1}\left(P\right)=\frac{1}{2\pi }\sqrt{\frac{3\left[S+N(P)\right]}{\rho AL^{2}}}.$$

The considered structure may not be a traditional tensegrity structure, as it is stable only under tensile forces [23]. However, its behavior serves as a representative model to understand the behavior of tensegrity structures. It allows us to analyze the influence of the initial prestress level S on the unstable regions, which is the focus of this study. In this case, Equation (16) is as follows:

(23) $$k_{L}+\beta k_{G}-\frac{\theta^{2}}{4}m=0;\;\beta =\left(1\pm \frac{1}{2}\upsilon \right).$$

This equation helps to determine the boundaries of the first instability regions in the parameter plane described by parameters (17):

(24) $$\eta =\frac{1}{\pi }\sqrt{\frac{k}{m_{\rho }}};\;k=\beta \left[k_{G}\left(S\right)+k_{GN}\left(N\right)\right].$$

For the time-independent load ${(P}_{t}=0\to \upsilon =0)$, the resonant frequency (24) depends solely on the constant part P of the load and is twice the free frequency (22)_2_.

The stiffness $k_{GN}\left(N\right)$ depends on the axial force N, which is a result of the load P. It can be determined through the static equilibrium of node 2 in the actual configuration (Figure 1c). The axial force N is calculated as follows:

(25) $$N=\frac{P}{2\sin\alpha }-S;\;\sin\alpha =\frac{q}{\sqrt{L^{2}+q^{2}}}.$$

The displacement q can be obtained using either the quasi-linear (2nd order theory) or non-linear (3rd order theory) theories. In the quasi-linear theory, the displacement is given by:

(26) $$q=\frac{PL}{2S},$$

which tends to infinity $q\left(S=0\;kN\right)=\infty$ when there is no initial prestress (S=0 kN). In non-linear theory, the displacement is determined by solving the following equation:

(27) $$\frac{1}{L}\left(2S+\frac{EA}{L^{2}}q^{2}\right)q=P.$$

In the absence of initial prestress (S=0 kN), the displacement is given by the following formula:

(28) $$q(S=0)=\sqrt[{3}]{\frac{P}{EA}}L.$$

To illustrate the influence of external loads on the behavior of the structure, specific parameters are given. The cables are assumed to have a length of L=1 m and a diameter of ϕ=20 mm ($A=3.14\cdot 10^{-4}\;m^{2}$). The material properties of the cables were specified as follows: Young modulus E=210 GPa and density $\rho =7860\;kg/m^{3}$. Two constant load values P are considered: P=1 kN and P=5 kN. The range of the pulsatility index (17)_1_ is set between 0 and 0.75. The load capacity is assumed to be $N_{Rd}=110.2\;kN$ [31], ensuring that it does not exceed 85% of its maximum value ($S_{max}=70\;kN)$ with the range of initial prestressing forces S.

**Figure 2:**
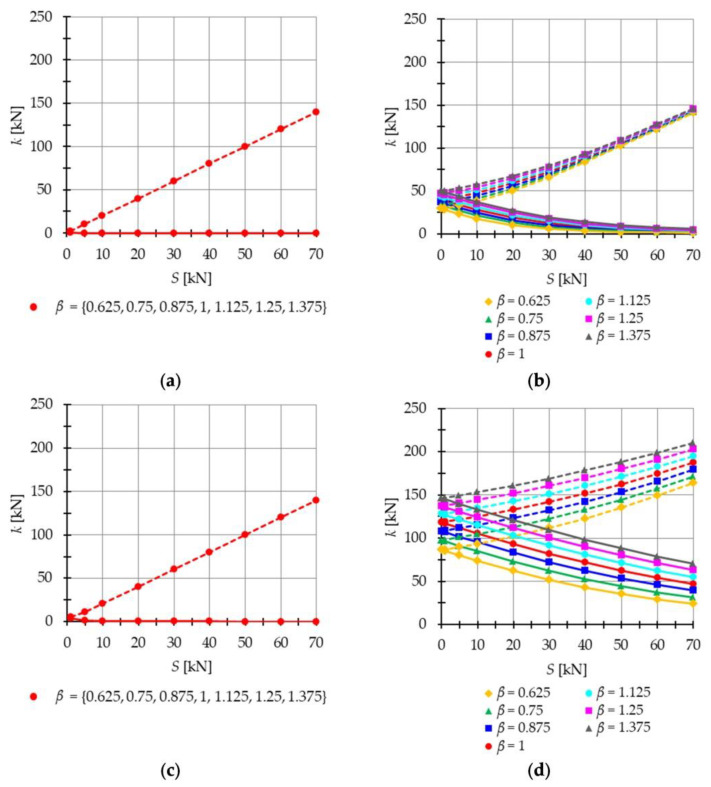
Influence of the initial prestress level S on the stiffness k (23) in the case of P=1 kN (**―** 1 kN, **- - -** 1 kN+S): (**a**) 2nd order theory, (**b**) 3rd order theory, and in the case of P=5 kN (**―** 5 kN, **- - -** 5 kN+S): (**c**) 2nd order theory and (**d**) 3rd order theory.

**Figure 3:**
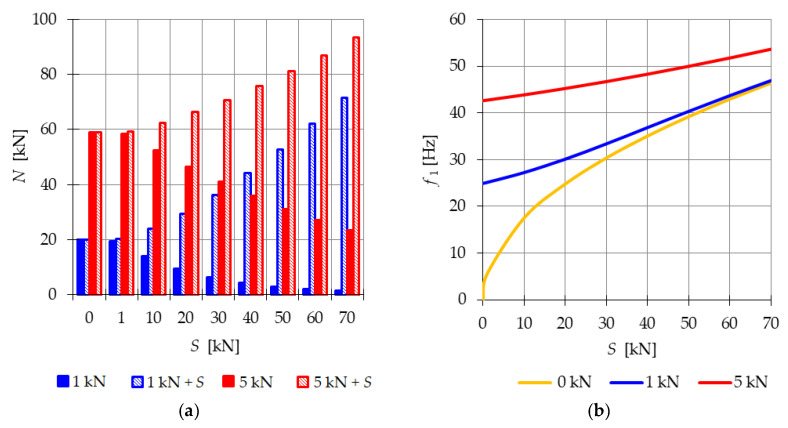
Impact of the initial prestress level S on: (**a**) the axial force N, (**b**) the first natural $f_{1}(0\;kN)$, and free frequencies $f_{1}\left(1\;kN\right)$, $f_{1}(5\;kN)$.

The influence of initial prestress level S on the stiffness k (24)_2_ is presented in Figure 2 (the stiffness resulting from the load P is marked with a solid line **―**, while the stiffness generated by the combined effect of the load and prestress forces P+S is marked with a dashed line **- - -**). Both the 2nd order (Figure 2a,c) and 3rd order (Figure 2b,d) theories are used. As can be seen, the quasi-linear theory is not appropriate to calculate the stiffness for structures characterized by a self-stress state and an infinitesimal mechanism. The graphs in Figure 2a,c coincide, which means that the stiffness does not depend on the load, which is not accurate. This approach neglects an important feature of tensegrity structures, namely, the stiffening effect caused by external loads. The load, which induces displacements according to the form of the infinitesimal mechanism, generates additional prestress in the structure. Tensile forces in the cables lead to additional tensile forces in the cables themselves and compressive forces in the struts. For such regimes, the initial response alone cannot accurately determine the behavior of the structure. Figure 2b,d demonstrate that the influence of nonlinearity is most significant at low initial prestress forces and low load values. Additionally, at lower load values, the initial prestress has a greater impact on the overall rigidity of the structure. The external load induces additional tensile forces in the cables. However, upon introducing the initial prestress, the normal forces resulting from the external load gradually decrease, reducing their influence on the displacement. Figure 3a illustrates the variation in the normal forces arising from the load, $N\left(P\right),$ and the normal forces generated by the combined effect of the load and prestress forces, N(P+S).

The 2nd order theory, which does not consider the stiffening effect of the structure under external loads that induce displacements consistent with the infinitesimal mechanism, is limited in accurately capturing the behavior of the structure. Therefore, in the subsequent analysis, the non-linear theory based on the assumption of large displacements (3rd order theory) is employed to calculate the stiffness of the structure. Initially, the dynamic behavior of the structure is investigated when it is loaded solely by the constant part P of the time-dependent load (t) ${(P}_{t}=0\to \upsilon =0)$. The impact of the initial prestress S on the natural frequency $f_{1}(0)$ and free frequencies $f_{1}(P)$ is determined using Equation (22) (Figure 3b). The natural frequency varies within the range of $f_{1}(0)=0$ to $f_{1}(0)=44.6\;Hz$. In the case of the free frequency, the external load acts as a prestress on the structure. Figure 3 shows the variation in the normal forces resulting from the load, N(P), and the normal forces generated by the combined effect of the load and prestress forces, N(P+S). The initial dynamic response (at S=0 kN) corresponds to the values of the natural frequency at the following force levels: $N\left(P=1\;kN\right)=20.20\;kN$ and $N\left(P=5\;kN\right)=59.12\;kN$ (Figure 3a—S=0 kN). Although the external load prestresses the structure, the introduction of initial prestress gradually reduces the normal forces caused by the external load, thereby decreasing its influence on the frequency. The analysis reveals that the impact of the initial prestress on the first vibration frequency diminishes as the load value increases.

**Figure 4:**
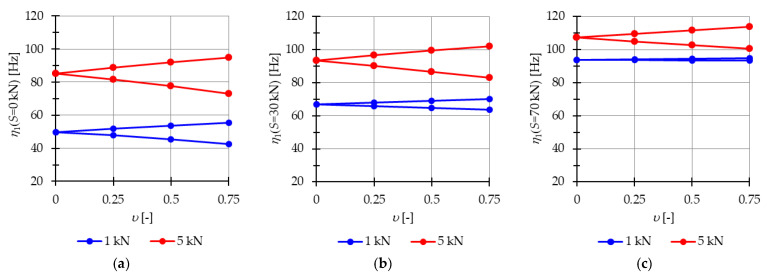
Limits of the main instability region of the two-element structure: (**a**) S=0 kN, (**b**) S=30 kN, (**c**) S=70 kN.

**Figure 5:**
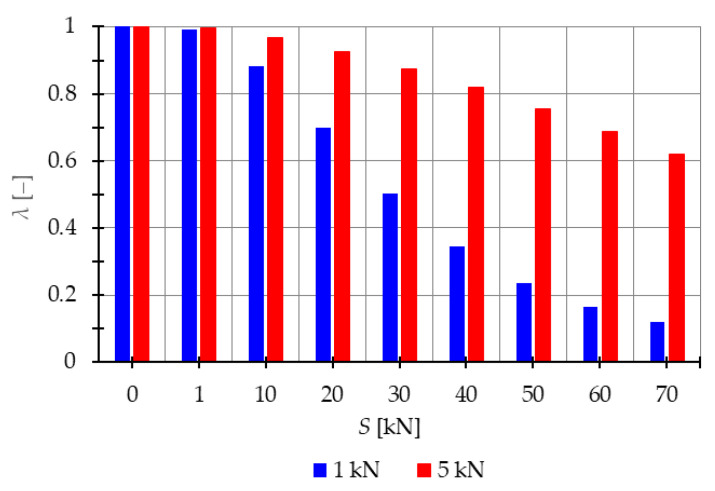
Influence of the initial prestress level *S* on the range of instability regions of the two-element structure.

In the subsequent analysis, the influence of the initial prestress S on the resonant frequencies η is examined when the periodic force with the constant part P, the amplitude $P_{t}$, and the frequency θ is applied. The impact of initial prestress S on the resonant frequencies η is investigated. Figure 4 illustrates the boundaries of the main instability region in the parameter plane of υ and η (17) as a function of the prestress level S. The results are presented for three cases of initial prestress: S=0 kN, S=30 kN, and S=70 kN. Since the structure exhibits only one infinitesimal mechanism, a single main instability region is determined. To quantify the changes in the area of the instability region, the nondimensional parameter λ (18) is calculated and depicted in Figure 5. This parameter provides a measure of the size of the instability region. By comparing the values of λ for different prestress levels, the influence of initial prestress on the extent of the instability region can be assessed.

The analysis reveals that as the initial prestress S increases, the resonant frequencies η also increase, and the range of the instability regions decreases. The level of initial prestress has a more significant impact on the extent of the instability regions when lower loads are applied. For example, when considering the load of P=1 kN, the parameter λ, which represents the size of the instability region, takes the values: λ=0.88 (at S=10 kN), λ=0.7 (at S=30 kN), and λ=0.12 (at S=70 kN). This means that the instability region is respectively 12%, 30%, and 88% smaller compared with the case where there is no initial prestress (S=0 kN). Similarly, for the load of P=5 kN, the instability is reduced by 3% (λ=0.97), 8% (λ=0.92), and 38% $\left(\lambda =0.62\right)$, for prestress levels of S=10 kN, S=30 kN, and S=70 kN, respectively.

Additionally, the extent of the instability regions is influenced by the level of the applied load. If the constant part of the load P increases, the area of the instability region also increases. For instance, in the case of the load P=5 kN the area of the instability regions is 1.09 times larger (at S=10 kN), 1.74 times larger (at S=30 kN), and 5.2 times larger (at S=70 kN) compared with the instability regions obtained for the load of P=1 kN.

The example presented in this study served to validate the proposed approach. The harmonic balance method was employed to derive formulas that enabled the determination of safe regions in the initial prestress function. These formulas provided valuable insights into the behavior and stability of tensegrity structures under varying levels of initial prestress. By utilizing the harmonic balance method, a comprehensive understanding of the safe areas in the prestress function was achieved, contributing to the assessment and design of stable tensegrity structures.

## 4. Tensegrity Structures

**Figure 6:**
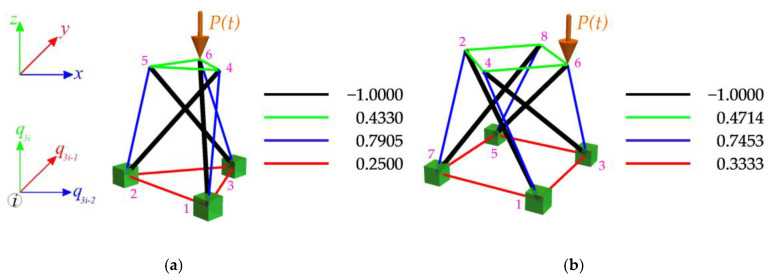
Values of the normalized self-stress states $y_{S}$: (**a**) Simplex module, (**b**) Quartex module [23].

The paper concentrates on dynamic stability analyses of tensegrity structures constructed using widely used tensegrity modules, namely the modified Simplex and Quartex modules. These modules are popular choices in the construction of tensegrity structures due to their versatility and adaptability. The analysis focuses on a linear connection with different ways of connecting modules in the structure. The structures are subjected to a force applied in the z-direction of one of the top nodes. The periodical loads P(t) are taken into account, and the influence of the initial prestress level S $\left(S=y_{S}S\right)$ on instability regions of the structures is considered. To perform the calculations, the eigenvectors $y_{S}$ obtained for a single module in Part 1 [23] are utilized. These eigenvectors represent the normalized self-stress states of the structure. Figure 6 illustrates the values of these normalized self-stress states, with the cables indicated in red, green, and blue and the struts shown in black. The calculations for determining the instability regions were carried out using a geometrically non-linear model implemented in a proprietary program written in the Mathematica environment.

The overall size of the modules is such that they can be contained within a cube where each side measures one meter. In the analysis, the structures are constructed using steel as the material, with a density of $\rho =7860\;kg/m^{3}$. The type A cables are composed of steel grade S460N and have a diameter of ϕ=20 mm. They are designed to have a load-bearing capacity of $N_{Rd}=110.2\;kN$ [31]. The Young modulus of the cables is E=210 GPa. The struts are composed of hot-finished circular hollow sections with a diameter of ϕ=76.1 mm and a thickness of t=2.9 mm. The struts are composed of steel grade S355J2, which has a Young modulus of E=210 GPa. The load-bearing capacity of the struts is $N_{Rd}=203.5\;kN$ [32] for the Simplex module and $N_{Rd}=193.9\;kN$ for the Quartex module [31]. In the analysis, the loads that cause displacements following the mechanism of the tensegrity structure are taken into consideration. This means that the applied loads are distributed and transmitted through the interconnected members of the structure, resulting in specific displacement patterns governed by the structural configuration. By studying the response of the tensegrity structure to these mechanism-induced loads, the analysis aims to explain how the structure behaves under realistic loading conditions and how it maintains its stability and integrity. This information is crucial for assessing the structural performance and ensuring the safe and efficient operation of tensegrity structures. For single modules, the minimum prestress value is assumed as $S_{min}=0\;kN$, while for structures, it depends on the type of the module. The maximum value is set as $S_{max}=110\;kN$. The main instability regions are determined by the pulsatility index (17)_1_, ranging from 0 to 0.75.

### 4.1. Tensegrity Single Modules

The first considered tensegrity structures are the single modified Simplex (Figure 6a) and Quartex (Figure 6b) modules. In these structures, all degrees of freedom (DoF) of the bottom nodes are fixed. For the Simplex module, nine DoFs are fixed, while for the Quartex module, twelve DoFs are fixed. Both modules are characterized by having one infinitesimal mechanism [1]. The modules are subjected to the periodic load P(t), which is applied to the 6th node (Figure 6). Two variants of the constant part P of the periodic force are considered, i.e., P=10 kN and P=20 kN.

**Figure 7:**
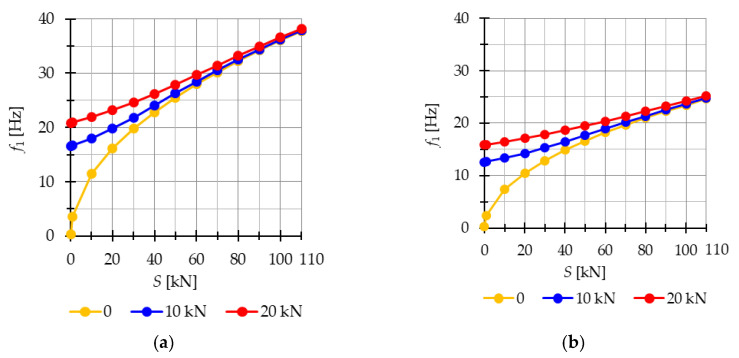
Impact of the initial prestress level *S* on the first natural $f_{1}\left(0\right)$ and free frequency $f_{1}\left(10\;kN\right),$ $f_{1}(20\;kN)$: (**a**) Simplex module, (**b**) Quartex module.

**Figure 8:**
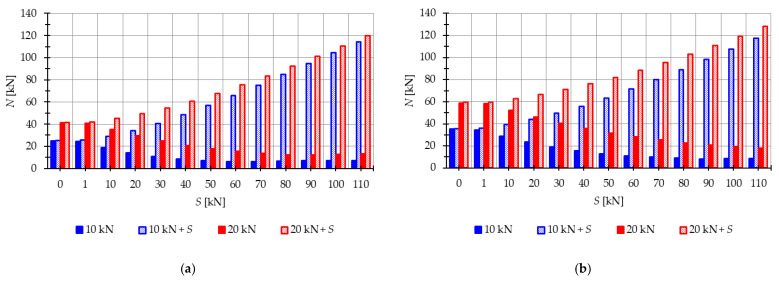
Impact of the initial prestress level *S* on the axial force N: (**a**) Simplex module, (**b**) Quartex module.

The natural $f_{1}(0)$ and free frequencies $f_{1}(P)$ of the single-mechanism modules are calculated to analyze their behavior under prestress. In the case of single-mechanism modules, there is only one frequency that depends on the level of prestressing. Figure 7 illustrates the influence of initial prestress S on the first frequency. For the Simplex module (Figure 7a), the natural frequency varies from 0 to 37.8 Hz, while for the Quartex module (Figure 7b), it ranges from 0 to 24.6 Hz. Considering the free frequencies $f_{1}\left(10\;kN\right)$ and $f_{1}(20\;kN$), the initial dynamic response at S=0 corresponds to the values of the natural frequency at the following force levels:

- For the Simplex module (Figure 8a): $N\left(P=10\;kN\right)=25.01\;kN$ and $N\left(P=20\;kN\right)=41.31\;kN$;
- For the Quartex module (Figure 8b): $N\left(P=10\;kN\right)=35.28\;kN$ and $N\left(P=20\;kN\right)=58.82\;kN$.

In both modules, the external load prestresses the structure. However, after introducing the initial prestress, the normal forces resulting from the external load progressively decrease, reducing their influence on the frequency. Figure 8 visualizes the change in the value of normal forces caused by the load N(P) and the normal forces generated by the combined action of the load and prestress N(P+S).

The analysis revealed that the Simplex module is more sensitive to changes in the level of prestress compared with the Quartex module. Both modules exhibit a significant influence of prestressing on the frequency and resonant behavior, especially at lower values of initial prestress forces. Similar to the two-element structure, the effect of prestressing diminishes as the load value increases.

**Figure 9:**
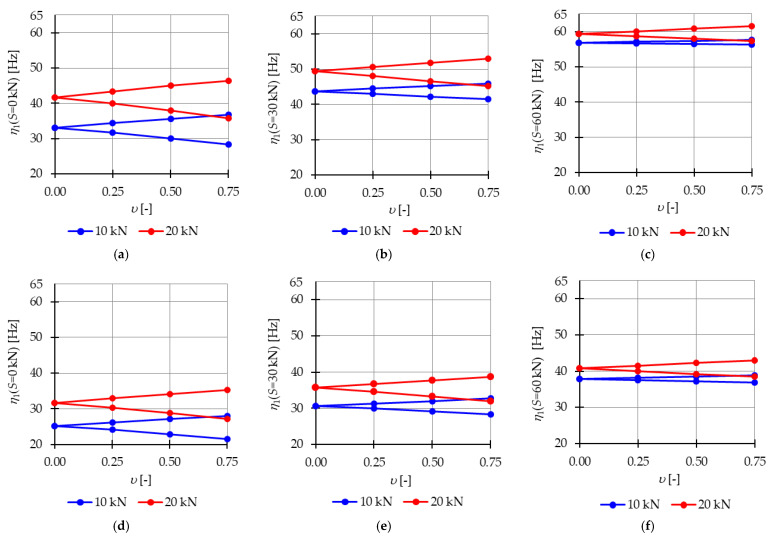
Limits of the main instability region of the Simplex module: (**a**) S=0 kN, (**b**) S=30 kN, (**c**) S=60 kN and of the Quartex module: (**d**) S=0 kN, (**e**) S=30 kN, (**f**) S=60 kN.

**Figure 10:**
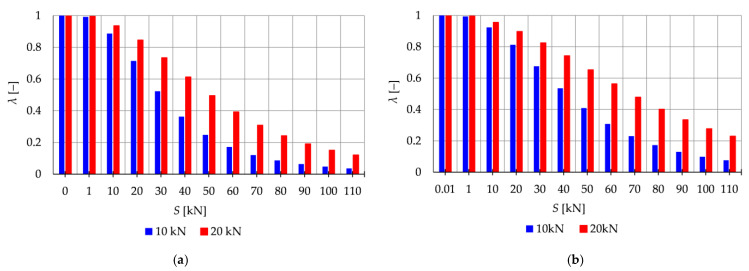
Influence of the initial prestress level *S* on the range of instability regions: (**a**) Simplex module, (**b**) Quartex module.

Figure 9 presents the limits of the main instability regions for different levels of initial prestress (S=0 kN, S=30 kN, and S=60 kN), while Figure 10 shows the nondimensional parameter λ (18), which measures the changes in the area of the instability regions. Increasing the prestress level leads to higher resonant frequencies and a reduction in the range of the instability regions. The introduction of self-stress significantly improves the stability of the structure.

For the Simplex module (Figure 9a–c and Figure 10a), at lower values of prestressing forces (S∈<0 kN;40 kN>), the parameter λ ranges from 1.0 to 0.36 for P=10 kN and from 1.0 to 0.61 for P=20 kN. This means that at S=40 kN, the instability region is only 64% and 39% smaller, respectively, than the area of the instability region at zero initial prestress. However, further prestressing considerably narrows the instability regions, and at the maximum level, λ ranges from 0.03 to 0.12, resulting in a decrease of the instability region by 97% and 88%.

Similarly, for the Quartex module (Figure 9d–f and Figure 10b), at lower values of prestressing forces (S∈<0 kN;40 kN>), the parameter λ ranges from 1.0 to 0.53 for P=10 kN and from 1.0 to 0.74 for P=20 kN. This means that at S=40 kN, the instability region is only 47% and 26% smaller, respectively, than the area of the instability region at zero initial prestress. Further prestressing significantly narrows the instability regions, and at the maximum level, λ ranges from 0.07 to 0.23, resulting in a decrease of the instability region by 97% and 88%, respectively.

In summary, higher values of initial prestress reduce the risk of excitation of motion with increasing amplitudes over time, indicating improved stability of the tensegrity structure.

### 4.2. Tensegrity Towers

**Figure 11:**
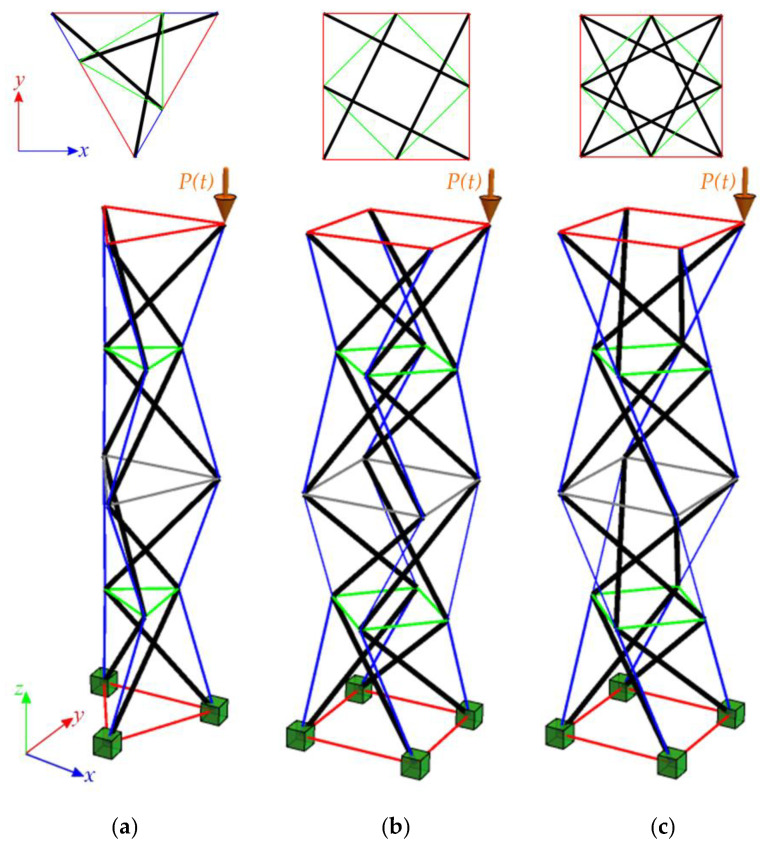
Linear four-module models: (**a**) S4, (**b**) Q4-connection A, (**c**) Q4-connection B. The colors are the same as in Figure 6.

The analysis focuses next on structures built with linearly connected modules, referred to as towers. The Simplex modules can only be connected in one way (Figure 11a), while the Quartex modules can be connected in two ways (Figure 11b,c). The connection method for Quartex modules is important, as confirmed in Part 1. In the analysis of Quartex modules, two types of connections are considered: connection A and connection B. Connection A involves overlapping the struts in a plan view. This means that the struts intersect and overlap each other in the structure, creating a configuration where the struts cross each other at certain points. On the other hand, connection B is characterized by a star-like pattern formed by the struts. In this configuration, the struts radiate from a central point and do not intersect or overlap each other. In the previous part, an abnormality in the dynamic behavior of Quartex towers was observed. The number of natural frequencies, depending on the prestressing, should be equal to the number of infinitesimal mechanisms. This principle holds true for Simplex towers. However, in the case of Quartex towers constructed with four or more modules, there is an additional frequency that depends on the initial prestress. This frequency is non-zero in the absence of initial prestress (S=0 kN) and its value varies with the change in prestress. Therefore, the analysis considers structures built with three (S3, Q3-A, Q3-B) and four (S4, Q4-A, Q4-B) modules. Similar to previous cases, all degrees of freedom of the bottom nodes are fixed, resulting in nine fixed degrees of freedom for Simplex structures and twelve fixed degrees of freedom for Quartex structures. The structures are characterized by three and four infinitesimal mechanisms for structures built with three and four modules, respectively. The structures are loaded with the periodic load P(t) applied to one of the upper nodes. In this analysis, the constant part of the periodic force is set as P=5 kN.

#### 4.2.1. Tensegrity Three-Module Towers

**Figure 12:**
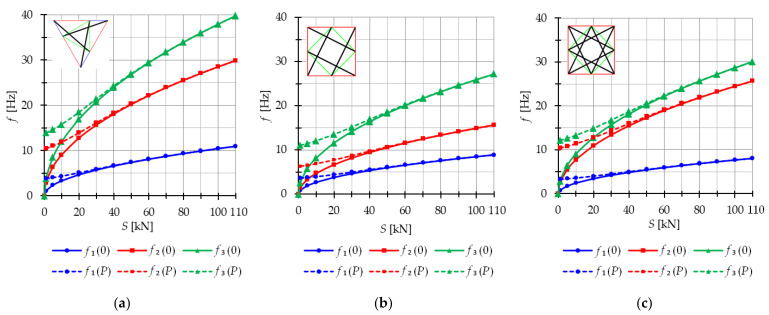
Impact of the initial prestress level *S* on the natural $f_{i}(0)$ and free frequencies $f_{i}\left(P\right)$: (**a**) S3, (**b**) Q3-A, (**c**) Q3-B.

The analysis focuses next on three-module tensegrity towers, specifically the S3, Q3-A, and Q3-B structures. The minimum level of self-stress for all towers is equal to $S_{min}=1\;kN$. These towers are characterized by three infinitesimal mechanisms [1] and behave typically as structures characterized by infinitesimal mechanisms, i.e., the number of natural frequencies depending on the prestressing is equal to the number of infinitesimal mechanisms. In the absence of initial prestress (S=0 kN), the natural frequencies are zero, and their values vary with the change in prestress. The impact of initial prestress S on the natural frequencies $f_{i}(0)$ is shown in Figure 12. The values of the three natural frequencies vary within the range of

- 0 to 10.96 Hz ($f_{1})$, 29.92 Hz ($f_{2})$ and 39.81 Hz ($f_{3})$—for the Simplex tower (Figure 12a);
- 0 to 8.91 Hz ($f_{1})$, 15.66 Hz ($f_{2})$ and 27.19 Hz ($f_{3})$—for the Quartex tower with connection A (Figure 12b);
- 0 to 8.06 Hz ($f_{1})$, 25.68 Hz ($f_{2})$ and 30.1 Hz ($f_{3})$—for the Quartex tower with connection B (Figure 12c).

In all cases, the frequencies increase with the rise of the initial prestress, and higher frequencies are more sensitive to the change in prestress. Generally, the natural frequencies of the structure built with the Simplex modules (S3) are higher than those of the structure built with the Quartex modules (Q3-A, Q3-B), which is consistent with the behavior observed in the single module case. The impact of initial prestress S on the free frequencies $f_{i}(P)$ is shown in Figure 12. In the absence of initial prestress (S=0 kN), the free frequencies are equal:

- $f_{1}(0)=3.83\;Hz$, $f_{2}(0)=10.42\;Hz$ and $f_{3}\left(0\right)=13.89\;Hz$—for Simplex tower (Figure 12a);
- $f_{1}(0)=3.63\;Hz$, $f_{2}(0)=6.31\;Hz$ and $f_{3}(0)=11.05\;Hz$—for Quartex tower with connection A (Figure 12b);
- $f_{1}(0)=3.28\;Hz$, $f_{2}(0)=10.41\;Hz$ and $f_{3}(0)=12.16\;Hz$—for Quartex tower with connection B (Figure 12c).

The application of the external load increases the vibration frequencies, but at approximately S=40 kN, the free frequencies become equal to the natural frequencies for all towers. The Q3-B model behaves similarly to the S3 model, where the second frequency is closer to the third frequency. On the other hand, for the Q3-A model, the second frequency is closer to the first frequency.

**Figure 13:**
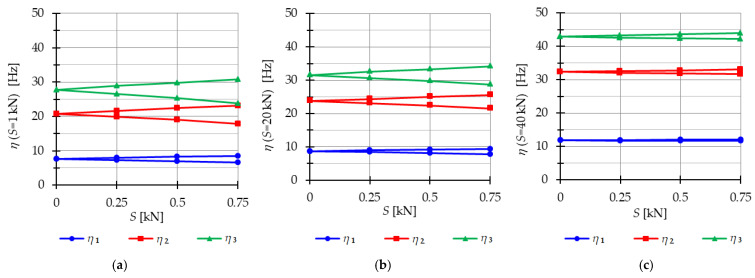
Limits of the main instability region of the Simplex tower (S3): (**a**) S=1 kN, (**b**) S=20 kN, (**c**) S=40 kN.

**Figure 14:**
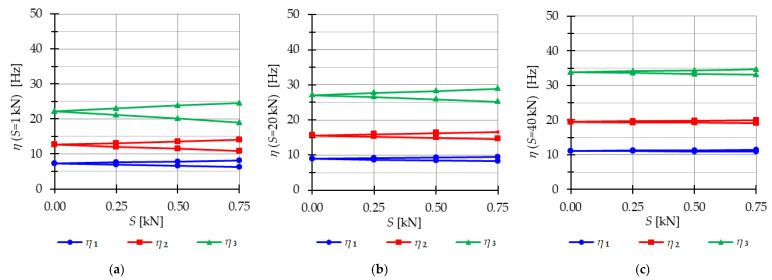
Limits of the main instability region of the Quartex tower with connection A (Q3-A): (**a**) S=1 kN, (**b**) S=20 kN, (**c**) S=40 kN.

**Figure 15:**
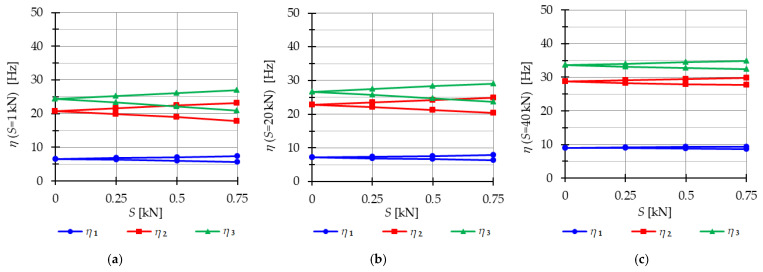
Limits of the main instability region of the Quartex tower with connection B (Q3-B): (**a**) S=1 kN, (**b**) S=20 kN, (**c**) S=40 kN.

**Figure 16:**
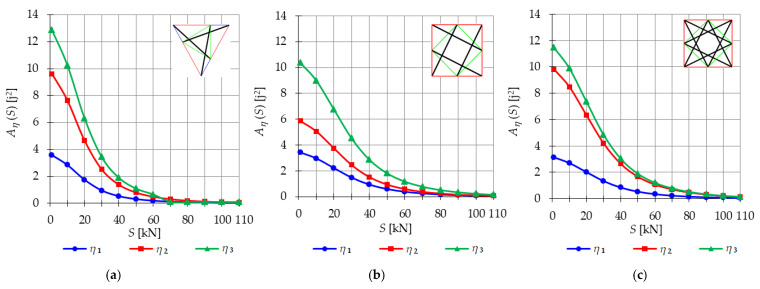
Influence of the initial prestress level *S* on the area of unstable regions $A_{\eta }(S)$ of three-module towers: (**a**) S3, (**b**) Q3-A, (**c**) Q3-B.

The analysis indicates that the Simplex tower is more sensitive to changes in the level of prestress compared with the Quartex towers. The influence of prestressing on frequency is most significant at low values of initial prestress forces, which are consistent across all towers examined. Similar conclusions can be drawn for the resonant frequencies. Figure 13, Figure 14 and Figure 15 show the limits of the main instability regions for three cases of initial prestress (S=1 kN, S=30 kN, and S=60 kN). Since the structures have three infinitesimal mechanisms, three main regions of instability are determined, corresponding to three resonant load frequencies: η1,η2, and η3. The area of instability regions is larger at higher frequencies. Regarding free frequencies, the resonant frequencies exhibit a similarity between the Q3-B and S3 models. The locations of the instability regions in these two towers show that the second and third instability regions are close to each other and partially overlap at lower levels of the self-stress state. This behavior is considered dangerous for the structure because it increases the probability of resonance occurring. In contrast, for the Q3-A model, the instability regions do not overlap. To compare the behavior of the towers, the influence of the initial prestress S on the area of instability regions $A_{\eta }(S)$ is shown in Figure 16. The area of the instability regions is similar for the Q3-B and S3 models, indicating a comparable response to prestress.

**Figure 17:**
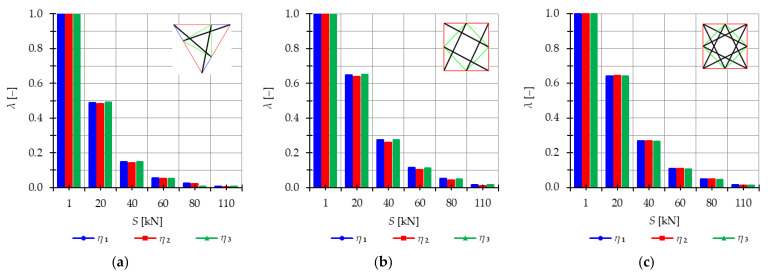
Influence of the initial prestress level *S* on the range of instability regions of three-module towers: (**a**) S3, (**b**) Q3-A, (**c**) Q3-B.

The nondimensional parameter λ (18) is calculated to measure the changes in the area of the instability regions, as shown in Figure 17. Similar to the previous example, as the level of initial prestress S increases, the resonant frequencies increase while the range of the instability areas decreases. This trend is consistent across all cases. At low values of initial prestressing forces, the range of the three main instability ranges changes slightly. For example, at S=10 kN, the parameter λ is equal λ=0.79 for the Simplex tower and λ=0.86 for the Quartex towers. This means that the instability regions are, respectively, 21% and 16% smaller than the regions at the minimum level of initial prestress $(S_{min}=1\;kN$). However, further prestress significantly narrows the instability regions. At S=60 kN, λ=0.05 and λ=0.1 (the regions decrease by 98% and 90%), and at the maximum level—λ=0.01 (the region decreases by 99%). This indicates that the boundaries of instability regions practically coincide at high initial prestress levels.

The results obtained from the dynamic instability analysis confirm the conclusions from the static and dynamic analyses in Part 1 [1]. The impact of the load on the behavior of tensegrity structures is most significant at low initial prestress forces. The area of the main instability regions is closely related to the initial prestress, and the range of these regions decreases with increasing prestress level. This implies that the risk of unstable vibrations is higher at low levels of initial prestress. For all towers, the number of main instability regions depends on the number of infinitesimal mechanisms, which corresponds to the number of natural frequencies related to the initial prestress level.

#### 4.2.2. Tensegrity Four-Module Towers

**Figure 18:**
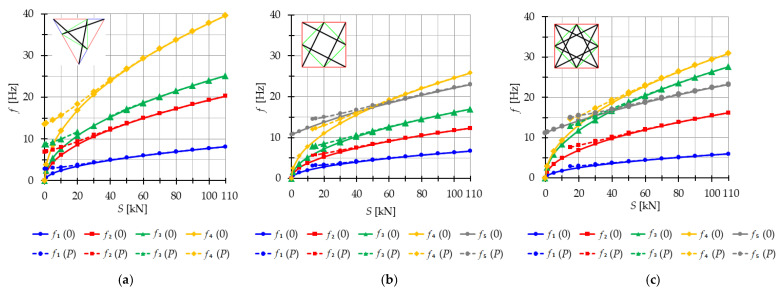
Impact of the initial prestress level *S* on the natural $f_{i}(0)$ and free frequencies $f_{i}\left(P\right)$: (**a**) S4, (**b**) Q4-A, (**c**) Q4-B.

**Figure 19:**
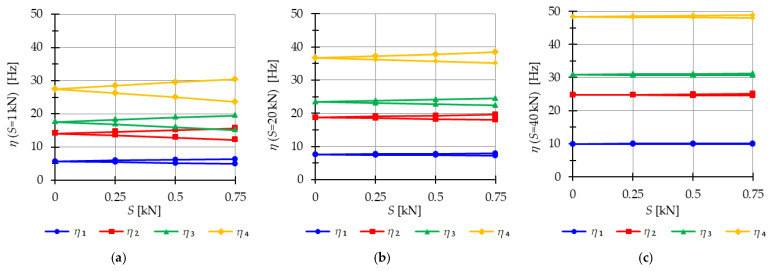
Limits of the main instability region of the Simplex tower (S4): (**a**) S=1 kN, (**b**) S=20 kN, (**c**) S=40 kN.

**Figure 20:**
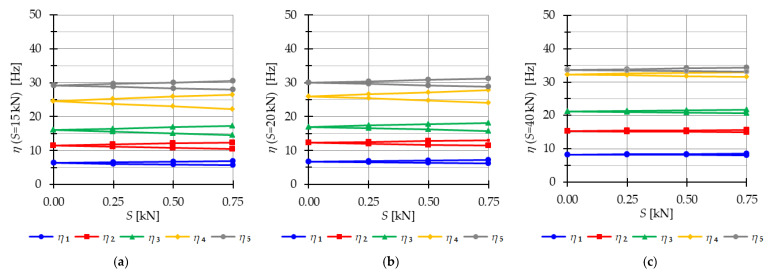
Limits of the main instability region of the Quartex tower with connection A (Q4-A): (**a**) S=15 kN, (**b**) S=20 kN, (**c**) S=40 kN.

**Figure 21:**
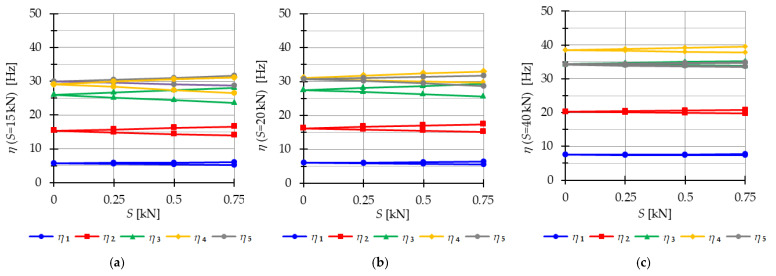
Limits of the main instability region of the Quartex tower with connection B (Q4-B): (**a**) S=15 kN, (**b**) S=20 kN, (**c**) S=40 kN.

The analysis focuses next on four-module tensegrity towers, namely S4, Q4-A, and Q4-B. In Figure 18, the natural frequencies $f_{i}(0)$ and the free frequencies $f_{i}(P=5\;kN)$ are shown, while in Figure 19, Figure 20 and Figure 21, the instability regions are visualized. It is important to note that these towers have a minimum level of self-stress that differs for each structure: $S_{min}=1\;kN$ for S4, $S_{min}=13\;kN$ for Q4-A, and $S_{min}=15\;kN$ for Q4-B (for comparison purposes, $S_{min}=15\;kN$ is used for both Quartex towers). All considered towers are characterized by four infinitesimal mechanisms [1]. The Simplex tower (S4) behaves typically as a tensegrity structure, where the number of frequencies depending on the prestress is equal to the number of infinitesimal mechanisms (Figure 18a). However, in the case of Quartex towers (Q4-A, Q4-B), there is an additional frequency that is dependent on the initial prestress. This natural frequency is not zero in the absence of initial prestress (S=0 kN), and its value varies with changes in prestress (Figure 18b,c).

Comparing the frequencies of three-module and four-module structures, the number of frequencies related to the initial prestress level changes, but the highest frequency associated with the initial prestress level remains the same for the corresponding structure (compare Figure 12 and Figure 18). For example, comparing the third frequency of the three-module Simplex tower and the fourth frequency of the four-module Simplex tower, they are practically equal. The range of these frequencies changes slightly, such as from 13.9 Hz to 39.8 Hz for the three-module structure and from 13.72 Hz to 39.58 Hz for the four-module structure.

The Quartex towers introduce an additional fifth frequency that occurs due to the presence of the fourth module. This frequency is not zero in the absence of initial prestress (S=0 kN) and exhibits a more linear variation as the initial prestress level increases. Consequently, for the maximum assumed prestress level, the fifth frequency is lower than the fourth frequency for Q4-A and lower than the third frequency for Q4-B. This behavior is also reflected in the areas of the instability regions, where the fifth region of instability overlaps with other regions (Figure 20 and Figure 21), unlike the “normal” frequencies.

The considerations made for the four-module structures align with the previous deliberations on the three-module towers. The instability regions also overlap for the S4 (Figure 19) and Q4-B (Figure 20) models. For the model Q4-B, the instability regions coincide for the entire prestress range, while for the S4 model, they coincide for lower levels of initial prestress.

## 5. Conclusions

The work is devoted to the parametric analysis of tensegrity structures subjected to periodic loads. The regions of dynamic instability are initially determined for a simple two-element truss. Subsequently, attention is shifted to the structures constructed using tensegrity modules, i.e., the modified Simplex and Quartex modules. Two methods of connection, labeled A and B, are considered. In connection A, the struts overlap in a plan view, while in connection B, they form a star configuration. The influence of the level of prestress is taken into account in the presented research. The work completes Part 1 [23] of the research, and both papers constitute the complete procedure of the investigation of the dynamic analysis of tensegrity structures.

This research aims to fill a gap in the existing literature by investigating how tensegrity structures respond to periodic loads. The main goal is to identify the resonant frequencies of periodic excitations and determine unstable regions based on the initial prestress level. In terms of physical interpretation, unstable regions indicate that the structure experiences vibrations with amplified amplitudes when subjected to specific load parameters. In this study, the harmonic balance method is employed as a powerful tool for conducting the dynamic stability analysis of tensegrity structures. The harmonic balance method is a well-established approach widely used in structural dynamics to analyze nonlinear systems subjected to periodic excitations. By utilizing this method, the study enables the identification of instability regions within the tensegrity structures being studied.

To validate the proposed approach, the two-element truss is examined as a case study. The harmonic balance method is applied to derive formulas that allow for the determination of safe regions in the initial prestress function. These formulas provide valuable insights into the behavior and stability of tensegrity structures under varying levels of initial prestress. By employing the harmonic balance method, a comprehensive understanding of the safe areas in the prestress function is achieved, which contributes to the assessment and design of stable tensegrity structures. The two-element truss is not a tensegrity structure, but it illustrates the behavior of tensegrity systems and allows deriving the explicit formulas of frequencies in the function of the initial prestress. The considerations serve as an explanation of the use of non-linear analysis in the dynamic analysis of tensegrity systems that stiffen under the application of an external load, and their initial response cannot be used to assess the behavior of the structure.

Regarding the behavior of the single tensegrity modules, the Simplex module is more prone to changes in the level of initial prestress. The introduced initial prestress improves the stability of the structure, and the impact of prestress is more significant at lower external loads.

In the case of towers, four-module structures constructed with Quartex modules exhibit abnormal behavior. For three-module structures, the number of instability regions is the same as the number of frequencies that depend on the level of initial prestress, as expected for tensegrity systems. However, for four-module structures, an additional frequency is identified. In contrast to the “normal” frequencies, this additional frequency is not zero in the absence of self-stress and changes more linearly. Towers built with the Simplex module, the Quartex module, and connection B perform more dangerous behaviors due to the overlapping of the instability regions, increasing the risk of resonance. Towers built with the Quartex module and connection A perform better with dynamic parameters as the instability regions do not overlap. The increase in initial prestress also improves the dynamic behavior of the structures considered.

The dynamic stability analysis reveals that the main instability regions are closely related to the initial prestress. The number of the main instability regions depends on the number of natural frequencies associated with this state, which is not always equal to the number of mechanisms. The range of the instability regions is closely related to the level of prestress, with the largest region observed at the minimum prestress level. As the prestress increases, the extent of the instability regions decreases. This indicates that the risk of exciting unstable vibrations is higher at lower levels of initial prestress.

The results obtained from the dynamic stability analysis confirm the conclusions obtained from the static and dynamic analyses, highlighting that the impact of load on the behavior of tensegrity structures is most significant at low values of initial prestress forces.

## Data Availability

The data presented in this study are available within the text of the paper.
